# Ultrasound-guided mini-incision technique for CHIVA

**DOI:** 10.1590/1677-5449.202201572

**Published:** 2024-10-07

**Authors:** Felipe Puricelli Faccini, Marcelo Halfen Grill

**Affiliations:** 1 Clínica Pró-Safena, Porto Alegre, RS, Brasil.; 2 Instituto de Cardiologia, Porto Alegre, RS, Brasil.; 3 Centro de Estudos Hiroshi Miyake, São Paulo, SP, Brasil.

**Keywords:** ultrasound-guided, CHIVA, venous operation, saphenous vein sparing, mini-incision, punção guiada por eco, CHIVA, safena, preservação, mini-incisão

## Abstract

CHIVA (*Cure Conservatrice et Hemodynamique de l'Insuffisance Veineuse en Ambulatoire*) is a surgical technique for varicose veins that avoids destroying the saphenous vein and collaterals. In some patients, the flush ligation of saphenous collaterals performed in the CHIVA technique may require more dissection than is desirable. This is especially valid for obese patients and thigh ligations. We report an innovative technique that uses duplex-guided puncture to bring the collateral closer to the skin and reduce the need for dissection and incision. The technique does not change any of the CHIVA principles. While the long-term results are not yet known, we expect them to be similar to normal CHIVA procedures.

## Resumo

CHIVA (*Cure Conservatrice et Hemodynamique de l'Insuffisance Veineuse en Ambulatoire*) é uma técnica de cirurgia de varizes que evita a destruição da safena e das colaterais. A ligadura rasante de colaterais da safena usando a técnica CHIVA pode requerer mais dissecção do que o desejado em alguns subgrupos de pacientes, principalmente os obesos. Este estudo descreve uma técnica inovadora de ligadura das colaterais com mini-incisões guiadas por eco-Doppler. A técnica consiste em puncionar com eco e passar um fio ao redor da colateral para puxá-la para cima. Uma pequena incisão é feita exatamente onde a colateral está situada sob a pele. A veia é tracionada e ligada em sua junção com a safena. A técnica é factível e pode diminuir a incisão e o trauma cirúrgico. Os resultados a longo prazo são desconhecidos, mas esperamos que sejam semelhantes aos da técnica CHIVA, já que nenhum dos preceitos é modificado.

## INTRODUCTION

CHIVA is the French acronym for “Cure conservatrice et Hemodynamique de l’Insuffisance Veineuse en Ambulatoire” (Conservative and Hemodynamic Treatment of Venous Insufficiency in the Office). It is a saphenous-sparing therapeutic approach for lower limb chronic venous disease (CVD) based on hemodynamic concepts proposed by Claude Franceschi in 1988.^_1_^ The rationale behind this hemodynamic approach is that increased transmural pressure (TMP) is responsible for the progression of the signs and symptoms of CVD, such as varicosities, edema, pain, itching, dermatitis, and ulcers. TMP is increased in superficial venous disease because of the higher hydrodynamic pressure caused by the absence of dynamic pressure fractioning and the presence of closed shunts (deep to superficial). We basically ligate connections that transfer pressure to other veins. There are 4 important points in CHIVA. First, we should eliminate the source of reflux (escape point), for example ligating a pelvic leak or doing a crossotomy. Second, we preserve as many veins as possible (mainly the re-entry perforators), since excessive ligation may result in saphenous phlebitis and loss of the vein with its consequences. The leg perforators are more commonly a manifestation of re-entry of blood that has escaped above than of the disease itself. Third, we should eliminate veins left without drainage to avoid superficial phlebitis of the collaterals. Lastly, we should fraction the blood column if it is too long.

One advantage of this technique is preservation of saphenous and venous leg capital. This is useful for coronary and arterial bypasses or natural bypass in case of a deep vein thrombosis and also stimulates less vein remodeling.^_2_^ Obese patients and deep collaterals pose difficulties for this technique because the incisions required to perform flush ligation are more difficult and create more trauma than is desirable. Surgical experience may minimize this surgical trauma, but depending on the patient, the surgical trauma is unavoidable.

Some authors advocate performing the treatment with endolaser. In our opinion, it is difficult to precisely burn the saphenous vein at the saphenous-collateral and saphenous-femoral junctions using endolaser, and leaving a stump may compromise results. Segmental fractioning of the saphenous vein can also be achieved using endolaser.^_3_^ We consider that these alternatives may be excellent, but they add variables to the procedure that will take years to be validated in clinical trials.

The current doctor/patient trending topic is minimizing incisions and cosmetic concerns cannot be overlooked. We have developed a simple technique that incurs no extra cost and can minimize incisions and surgical trauma, adapting CHIVA to this new trend. We can achieve better cosmetic results, especially in patients with deeper collaterals and higher body mass index. There is no change to the CHIVA technique and we therefore expect similar long-term results. If we can offer patients even smaller incisions than the usual CHIVA incisions, we may be able to offer an even more cosmetic method that preserves the saphenous vein. This is important because of the current trend of saphenous overtreatment.^_4__,__5_^

## THE TECHNIQUE

**Figure 1:**
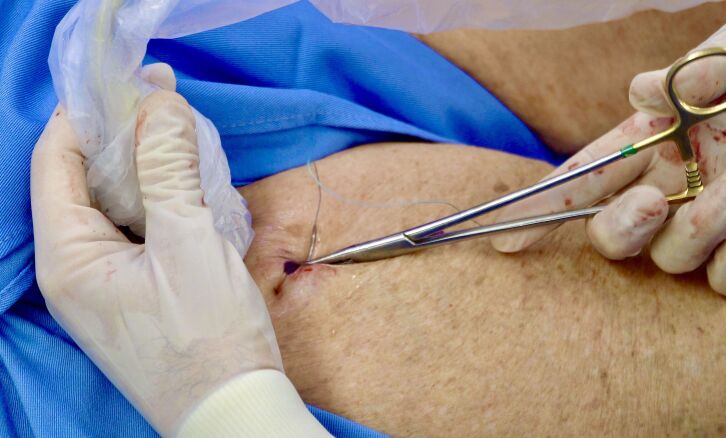
The needle is passed behind the vein with real-time visualization using Duplex scanning.

**Figure 2:**
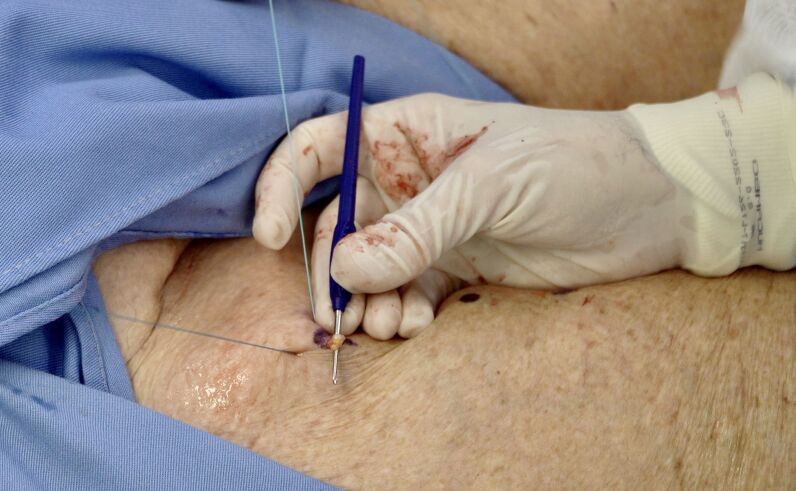
After a small incision, the vein is individualized.

**Figure 3:**
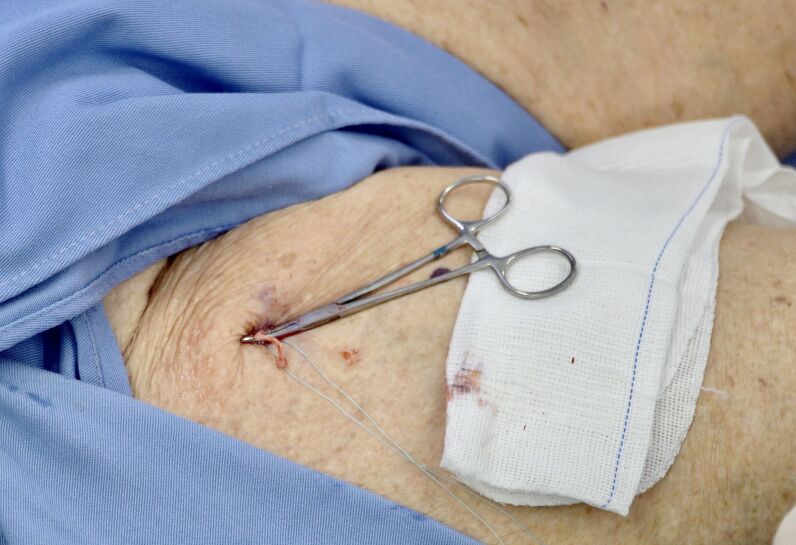
The vein is ligated close to the junction with the saphenous vein in order not to leave a stump.

We perform a CHIVA duplex scan before the operation and mark the skin for incisions as usual.^_1_ _,6_^ Before the operation, we prepare the patient and the duplex probe to be used during the procedure. B mode is used to visualize the great saphenous vein and the collateral that we have planned to ligate. Local anesthesia is injected. A long needle is used to perforate the skin (out-in) half a centimeter lateral to the collateral, we pass it behind the vein under duplex guidance and perforate the skin (in-out) once more, exiting half a centimeter lateral of the other side of the collateral (Figure 1). The thread is then pulled tight on both sides to create a rigid wire pulling the vein close to the skin. The vein is then palpated against the wire and its position is confirmed with duplex ultrasound. A mini-incision of 3-4 mm is made just above the vein, which is followed to its connection with the saphenous vein (Figure 2). If necessary, we use ultrasound to confirm the distance from the saphenous vein. If the collateral is too enlarged, we may make a larger incision, but surgical trauma remains reduced. Usual non-absorbable flush ligation is performed (Figure 3). The skin is closed and the wire is pulled out through one of the sides. The video in the Supplementary Material shows how we do it step by step. We obtained informed consent from the patient and CONEP case report approval is not needed for this innovative technique description.

## CONCLUSIONS

CHIVA has made a considerable contribution to diminishing surgical trauma and remodeling compared to destructive methods. Sometimes the thigh incisions used for CHIVA need to be large due to patient characteristics. Some consider that this is a limitation of the technique. The mini-incision technique diminishes surgical trauma and allows us to make a smaller incision, closer to the size of an introducer sheath, as used in other techniques. The technique is feasible and can minimize surgical trauma even further than with the usual CHIVA. The hemodynamic procedure and CHIVA routine are maintained; thus, we expect similar long-term results.

The technique improves the cosmetic results of CHIVA, which have recently become an important worry for both patients and phlebologists. The most important aspect is that we may be able to improve cosmetic results without foregoing the advantages of CHIVA. The saphenous vein will still be available for bypass and to compensate trauma or thrombosis in the deep system. Furthermore, the long-term venous remodeling that happens after saphenous elimination may be prevented.

## Supplementary Material

Supplementary material accompanies this paper.

Supplementary Video MINI-CHIVA step by step.

This material is available as part of the online article from https://doi.org/10.1590/1677-5449.202201572
